# Serum extracellular vesicle microRNA dysregulation and childhood trauma in adolescents with major depressive disorder

**DOI:** 10.17305/bjbms.2022.7110

**Published:** 2022-06-03

**Authors:** Liu-Yi Ran, Yi-Ting Kong, Jiao-Jiao Xiang, Qi Zeng, Chen-Yu Zhang, Lei Shi, Hai-Tang Qiu, Chuan Liu, Lin-Li Wu, Ya-Lan Li, Jian-Mei Chen, Ming Ai, Wo Wang, Li Kuang

**Affiliations:** 1 Mental Health Center, University-Town Hospital of Chongqing Medical University, Chongqing, China; 2 Department of Psychiatry, The First Affiliated Hospital of Chongqing Medical University, Chongqing, China

**Keywords:** Extracellular vesicle, miRNA, childhood trauma, major depressive disorder, adolescent

## Abstract

Major depressive disorder (MDD) seriously endangers adolescent mental and physical health. Extracellular vesicles (EVs) are mediators of cellular communication and are involved in many physiological brain processes. Although EV miRNAs have been implicated in adults with major psychiatric disorders, investigation into their effects in adolescent MDD remains scarce. In discovery set, we conducted a genome-wide miRNA sequencing of serum EVs from 9 untreated adolescents with MDD and 8 matched healthy controls (HCs), identifying 32 differentially expressed miRNAs (18 upregulated and 14 downregulated). In the validation set, 8 differentially expressed and highly enriched miRNAs were verified in independent samples using reverse-transcription polymerase chain reaction (RT-PCR), with 4 (miR-450a-2-3p, miR-3691-5p, miR-556-3p, and miR-2115-3p) of the 8 miRNAs found to be significantly elevated in 34 untreated adolescents with MDD compared with 38 HCs and consistent with the sequencing results. After the Bonferroni correction, we found that three miRNAs (miR-450a-2-3p, miR-556-3p, and miR-2115-3p) were still significantly different. Among them, miR-450a-2-3p showed the most marked differential expression and was able to diagnose disease with 67.6% sensitivity and 84.2% specificity. Furthermore, miR-450a-2-3p partially mediated the associations between total childhood trauma, emotional abuse, and physical neglect and adolescent MDD. We also found that the combination of miR-450a-2-3p and emotional abuse could effectively diagnose MDD in adolescents with 82.4% sensitivity and 81.6% specificity. Our data demonstrate the association of serum EV miRNA dysregulation with MDD pathophysiology and, furthermore, show that miRNAs may mediate the relationship between early stress and MDD susceptibility. We also provide a valid integrated model for the diagnosis of adolescent MDD.

## INTRODUCTION

Major depressive disorder (MDD) in adolescents is a common psychological problem throughout the world, seriously affecting the quality of life and safety of sufferers [1]. An epidemiological study showed a lifetime prevalence of 11.0% for MDD in adolescents with a 12-month prevalence of 7.5% [2]. Adolescent MDD is often associated with psychiatric comorbidity, leading to a series of educational and social problems, as well as an increased risk of adverse psychosocial problems in adulthood [2-4]. More seriously, MDD is a primary risk factor for suicide [1]. At present, the precise pathogenesis of adolescent MDD remains largely unknown. A major clinical challenge is the lack of reliable biomarkers for the prediction of early-stage diagnosis. Effective detection of early-onset depression plays a significant role in controlling its progression in clinical practice. Early detection or prediction of the symptoms of depression enables early intervention and prevention and can thus effectively reduce both short- and long-term negative consequences, such as educational and social problems, as well as the risk of adverse psychosocial issues in adulthood, and even suicide.

Adverse childhood experience is a well-established risk factor for the development of a variety of mental illnesses, including depression and suicide [5]. A recent study showed that childhood trauma is associated with poor mental health in adolescence, including sleep problems and symptoms of anxiety and depression [6]. A national longitudinal study in adolescents found that subjects who had been maltreated had a higher risk of depression and suicide ideation in young adulthood than subjects who had no maltreatment [7]. A growing body of evidence indicates that early adverse experiences are linked to the changes in the structure and functioning of multiple brain regions [8-10]. Epigenetic mechanisms that regulate levels of gene expression through DNA methylation and non-coding RNAs appear to play a significant part in these changes [11,12]. The adolescent brain is more structurally plastic and more susceptible to the adverse influence of environmental factors through epigenetic modifications [13]. Notably, it has been demonstrated that epigenetic modifications such as DNA methylation and the action of microRNAs (miRNAs) are associated with a susceptibility to depression in adolescence [14,15]. Consequently, epigenetic modification has been identified as a regulatory mediator between environmental stressors and MDD [16].

Extracellular vesicles (EVs) are cell-derived small vesicles with diameter between approximately 40 and 160 nm that are released from the cell by the fusion of multivesicular bodies with the cell membrane [17]. They are present in biological fluids and can contain many biological components of the cell, including nucleic acids, proteins, and lipids [18]. EVs act as signal carriers for the transmission of information or the exchange of materials between the cells, mediating intercellular communication through cargo transportation (including nucleic acids and proteins) between neighboring or distant cells. This communication results in phenotypic changes in the target cell and may induce numerous physiological or pathological alterations in the body [19,20]. More importantly, EVs can cross the blood-brain barrier and may thus be potentially excellent biomarkers for neuropsychiatric disorders [21,22].

MiRNAs, short and highly conserved non-coding RNA molecules with an approximate length of 17-22 nucleotides, regulate gene expression by silencing translation, or degrading target mRNAs. They participate in various biological processes in the brain, including synaptic plasticity and neurogenesis [23]. Recent evidence from postmortem tissue has demonstrated the significance of brain-associated miRNAs for the pathogenesis of depression [24]. MiRNAs are found not only in saliva, urine, blood, and other biological fluids but are also abundant in EVs. MiRNAs within EVs tend to persist longer than those in other environments as the lipid bilayer of the EV protects them and prevents their degradation [25]. There is accumulating evidence that, in addition to cancer [26], miRNAs in circulating EVs are promising biomarkers for the diagnosis of major psychiatric disorders in adults, including MDD [27], bipolar disorder [28], and schizophrenia [29]. However, there are no reports on the relationship between EV miRNAs and MDD in adolescents.

In the present study, we first conducted genome-wide expression profiling of miRNAs in serum EVs of adolescent MDD patients and healthy control (HC) subjects. Candidate miRNAs in EVs were identified in an independent adolescent MDD-HC sample by quantitative reverse-transcription polymerase chain reaction (RT-PCR). Then, we analyzed the hypothesis that these EV miRNAs mediate the relationship between childhood trauma and the development of MDD in adolescence by epigenetic means. Finally, we developed a model for the diagnosis of MDD in adolescence with childhood trauma and the expression levels of miRNAs in EVs.

## MATERIALS AND METHODS

### Participants

The study recruited 96 participants in total. The study had a two-stage design: the discovery set included 9 patients and 8 controls, and the validation set included 39 patients and 40 controls. The two sets had the same inclusion criteria, exclusion criteria, and clinical evaluation, but were independent with no repetition. All the participants were aged between 13 and 18 years, with education levels of primary school or above.

All the patients were recruited from the Mental Health Center at the University-Town Hospital of Chongqing Medical University between November 2020 and July 2021. Inclusion criteria were: (a) all patients were interviewed with Mini-International Neuropsychiatric Interview for Children and Youth (MINI Kid) [30] and satisfied the criteria for MDD in the Diagnostic and Statistical Manual of Mental Disorders, IV edition; (b) in terms of assessments, scores on the Children’s Depression Rating Scale-Revised (CDRS-R) were over 40 [31], and on the Hypomania Check List-32 scores were <14 [32]; and (c) participants had not received any antidepressant therapy, including drugs, physical therapy, or psychotherapy. Exclusion criteria were: (a) patients with severe heart, kidney, endocrine, blood, autoimmune, and other internal diseases; (b) current and past brain organic diseases, history of seizures, and other severe mental disorders; and (c) abuse of alcohol or other psychoactive substances. The HC subjects consisted of age- and gender-matched adolescents who were recruited during physical examinations in the University-Town Hospital of Chongqing Medical University and the Health Recruitment. All controls had no diagnosis of any mental disorders and had no family history of mental illness, major somatic diseases, and neurodevelopmental disorders.

In the study, we used 17-item Hamilton Rating Scale for Depression (HAMD-17) [33], CDRS-R and The Patient Health Questionnaire 9 (PHQ-9) [34] to assess the severity of depression in patients. We use the General Anxiety Disorder 7 (GAD-7) [35] to assess symptoms of anxiety. The Children’s Global Assessment Scale (CGAS) [36] was used to assess the overall functioning of the children and the Childhood Trauma Questionnaire-Short Form [37] was used to detect early stressors in childhood. The specific assessment process is presented in the supplementary methods.

### Serum collection and EV isolation

About 6 ml of whole blood was collected from each participant in additive-free blood tubes. The blood was left to stand at room temperature for 1 h, after which it was centrifuged at 4°C for 10 min at 2000× *g*. The serum sample was obtained by centrifugation of the supernatant at 4°C for 10 min at 12,000× *g* and stored at −80°C. EVs were isolated from the serum utilizing the Total EV Isolation Kit (from serum, Invitrogen, USA, Catalog Number: 4478360), following the instructions (Supplementary Methods) provided by the manufacturer.

### EV verification

The characteristics of the isolated EVs were confirmed by transmission electron microscopy (TEM), protein marker (Western blotting), and nanoparticle tracking analysis detection (Supplementary Methods).

### Small RNA library construction and sequencing

RNA was extracted from the EV pellet using TRIzol™ LS Reagent (Invitrogen, 10296010) in accordance with the standard protocol. The concentration and size distribution of the isolated RNA were measured using the Qubit fluorometer (Thermo Fisher Scientific, Waltham, MA, USA) and Agilent 2100 Bioanalyzer (Agilent Technologies, USA). MiRNA library construction and sequencing were performed by GeneSky Biotechnologies (Shanghai, China). The library construction process is described in the supplementary methods. The sequencing of the library was performed using a paired-end sequencing strategy on an Illumina high-throughput sequencing platform (NovaSeq 6000).

### Bioinformatics analysis

The quality assessment of the raw data and alignments with reference sequences is described in the supplementary methods. The small RNA sequences from each sample were aligned with the miRNA precursor and mature sequence in the miRBase database using miRDeep2 v0.1.3 software to identify known small RNA species and expression. Differential expression of miRNAs between the two groups was performed using DESeq2 v1.10.1 software. The target genes of the differentially expressed miRNAs were predicted using the miRTarBase database (http://miRTarBase.cuhk.edu.cn/). The R package clusterProfiler v2.4.2 was used for Gene Ontology (GO) and Kyoto Encyclopedia of Genes and Genomes (KEGG) enrichment analyses of the target genes of the miRNAs, with the level of statistical significance at *p* < 0.05.

### Quantitative RT-PCR verification

Each of the serum EV samples was spiked into an equal amount of Caenorhabditis elegans microRNA (cel-miR-39, 1pmol, Ibibio, and miRB0000010-3-1) to monitor the RNA extraction efficiency. As an external control gene for expression quantification, cel-miR-39 normalized the target gene CT value of all test samples and reference samples. The procedure for EV RNA extraction was the same as that described above for sequencing. The total RNA was reverse-transcribed to cDNA using target miRNA-specific stem-loop reverse transcription primers (Supplementary Table 1 for primer sequences) and Reverse Transcriptase M-MLV (Takara, Dalian, China). Quantitative RT-PCR was conducted using miRNA-specific primers and SYBR® Premix Ex Taq™ II (Tli RNaseH Plus) (TaKaRa, Japan) on the ABI 7900 Real-Time PCR System (Thermo Fisher Scientific) following the manufacturers’ protocol. Each miRNA was amplified separately. Three technical replicates were used for each sample. After the RT-PCR reaction, the amplification and melting curves of the cDNA samples and the negative control were confirmed. We used the average CT values of the exogenous control gene (cel-miR-39) as the preprocessed observation object. We eliminated seven samples with outliers (five depressed and two healthy adolescents). The data quality control and preprocessing are presented in the supplementary methods. The 2^-ΔΔCt^ method was used to evaluate the quantitative RT-PCR results.

**TABLE 1:**
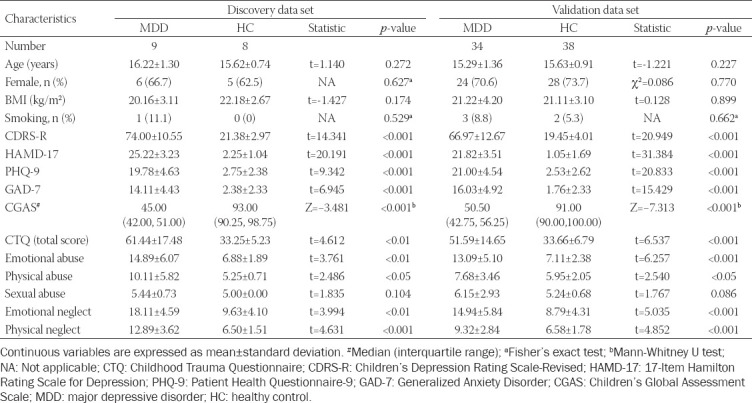
Participant characteristics

### Ethical statement

The study was approved by the University-Town Hospital of Chongqing Medical University Institutional Review Board (LL-202009). All participants and their parents or legal guardians received comprehensive information on the intention and course of the research and signed informed consent.

### Statistical analysis

Continuous data are described as means ± standard deviation (SD) or medians (interquartile range, IQR), while categorical data are shown as numbers (percentages).

Continuous data differences between the case and control groups were calculated using t-tests or the Mann–Whitney U test. The Chi-square test was applied for categorical data and Fisher’s exact test was used when appropriate (expected frequency <5). We conducted a principal component analysis (PCA) using the “prcomp” function in R software to aggregate the profiles of multiple miRNAs and used the first principal component as representative of each miRNA. Correlations between clinical symptoms and the relative expression levels of miRNAs were tested through Spearman rank analyses. Associations between differentially expressed miRNAs and adolescent MDD were assessed by binary logistic regression while adjusting for demographic and lifestyle (including age, gender, body mass index [BMI], and smoking status). The receiver operator characteristic (ROC) curve and area under the curve (AUC) were applied to examine the prediction accuracy of parameters for the disease risk. A forward stepwise method of logistic regression was conducted to recognize the best-fit integrated model. The mediating effects of miRNA were examined in PROCESS using least squares regression. The significance of mediating roles of miRNA was evaluated by the bootstrap method in the PROCESS program, by sampling 1000 samples with a 95% confidence interval (CI) for determination of effects [38,39]. SPSS 25.0 software was used for statistical analysis. Two-tailed *p* < 0.05 was considered to be statistically significant. The Bonferroni correction for multiple comparisons was used to identify statistically significant miRNAs, in which case *p* < 0.00625 (*p* = 0.05/8) was considered to be statistically significant.

## RESULTS

### EV miRNA discovery data set

The discovery set included nine untreated adolescent MDD patients and eight matched HC subjects. The characteristics of these participants are shown in Table 1. No statistically significant differences in age, gender, BMI, and smoking status were observed between the two groups.

**FIGURE 1:**
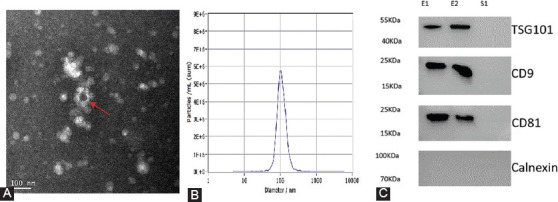
Identification of serum EVs. (A) Morphology of serum EVs was detected by transmission electron microscopy (100 nm). (B) EV particle size was calculated through nanoparticle tracking analysis. (C) The markers TSG101, CD9, CD81, and Calnexin were assessed by Western blotting. E1/E2: Two serum EV samples. S1: EV-depleted serum as negative control sample. EV: Extracellular vesicles.

Following the ISEV2018 guidelines [40], the EV characterization showed the presence of the EV-specific markers: CD9 molecule (CD9), CD81 molecule (CD81), and Tumor Susceptibility 101 (TSG101) in our serum. Calnexin was not detected in our serum samples. The size of the pellets ranged from 30 to 150 nm in diameter with a single peak at approximately 100 nm. The suspected EV structure was observed under TEM, as shown in Figure 1.

**FIGURE 2:**
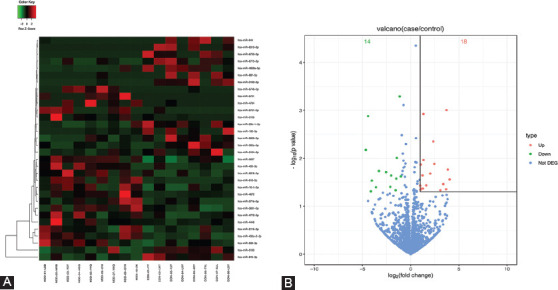
The miRNA expression profiles of serum EVs from depression patients and controls. (A) Heatmap of 32 differentially expressed serum EV miRNAs. (B) Volcano plot of differential miRNAs in serum EVs. The red dots display the upregulated expressed miRNA genes in case group relative to control group, and the green dots display downregulated miRNAs. The ordinate represents the P values of EV miRNAs. EV: Extracellular vesicles; miRNAs: microRNAs.

Differential analysis of miRNA expression in the EVs showed 32 differentially expressed miRNAs (*p* < 0.05, fold change ≥2) in adolescents with MDD compared to HC subjects. Of these, 18 miRNAs were upregulated and 14 miRNAs were downregulated (Figure 2 and Supplementary Table 2). To investigate the highly expressed miRNAs in the serum EVs, we selected the miRNAs with mean transcripts per million (TPM) >10 [29] resulting in the selection of 8 miRNAs in the EVs for quantitative RT-PCR verification in another independent sample (Supplementary Table 2).

**TABLE 2:**
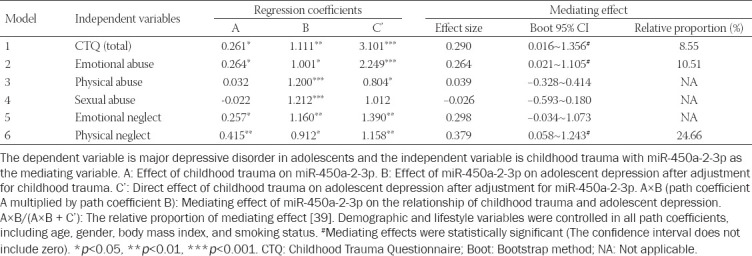
Mediating effect of miR-450a-2-3p in EVs on childhood trauma and adolescent depression

### EV miRNA verification by quantitative RT-PCR

The study included 34 participants with untreated MDD and 38 HC subjects (Table 1). No significant differences in demographics or lifestyle between the groups were found (*p* > 0.05). The MDD group scored significantly greater than the HC group on depression, anxiety, total childhood trauma, emotional abuse, physical abuse, emotional neglect, and physical neglect. The MDD group scored lower than the HC group on the global function score.

**FIGURE 3:**
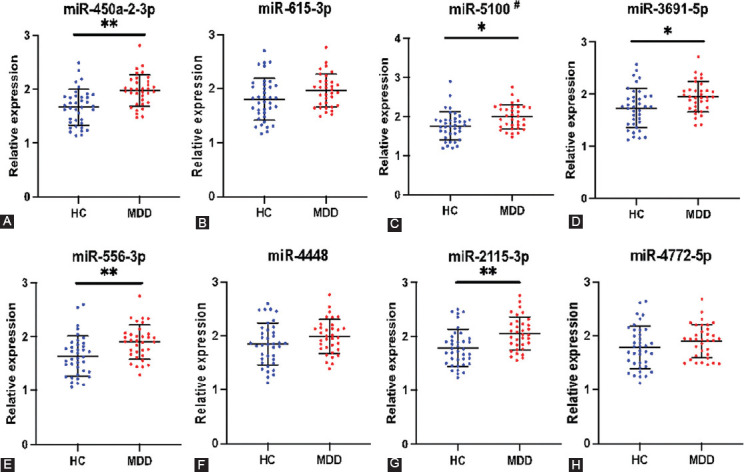
Verification of eight selected miRNAs in serum EVs by qRT-PCR. The ordinate displays the relative expressions of each miRNA measured by the 2^-ΔΔCt^ approach. #Contrary to the result of high-throughput sequencing. *Before the correction, p < 0.05. **After the Bonferroni correction, p < 0.00625. EV: Extracellular vesicles; miRNAS: microRNAs; HC: Healthy control; MDD: Major depressive disorder.

The RT-PCR verification demonstrated that the expression levels of five out of eight serum EV miRNAs were significantly elevated in the patients with adolescent MDD compared to the HC subjects (Figure 3). Among them, the expression patterns of four miRNAs (miR-450a-2-3p, miR-3691-5p, miR-556-3p, and miR-2115-3p) were consistent with the sequencing results, while the expression pattern of one miRNA (miR-5100) was opposite to the sequencing analysis (Figure 3). We then used the Bonferroni correction for multiple comparisons and found that three miRNAs (miR-450a-2-3p, miR-556-3p, and miR-2115-3p) still showed statistical significance (*p* < 0.00625) (Figure 3). After adjustments for demographics and lifestyle, we found that miR-450a-2-3p, miR-556-3p, and miR-2115-3p were significantly associated with depressed adolescents (*p* < 0.001, *p* < 0.01, and *p* < 0.01, respectively) (Supplementary Table 3).

Interestingly, correlation analysis indicated a high correlation (Spearman *r* > 0.85) between the expression levels of three miRNAs (*p* < 0.001), as shown in Supplementary Table 4. In addition, significant collinearity was observed between the three differentially expressed miRNAs (data not shown). We then conducted PCA on the three miRNAs and found that 95.62% of the variance was explained by PC-1, so the PC-1 was used for correlation analysis. The clinical correlation analysis showed a positive correlation between PC-1 and CDRS-R (*p* < 0.05), PC-1 and HAMD-17 (*p* < 0.01), PC-1 and PHQ-9 (*p* < 0.01), and PC-1 and GAD-7 (*p* < 0.01). It showed a negative correlation between PC-1 and the global function level (*p* < 0.01) (Supplementary Figure 1).

### Enrichment analysis of EV miRNA-targeted genes

To better understand the MDD pathogenesis, we then evaluated the potential target genes of the three miRNAs (miR-450a-2-3p, miR-556-3p, and miR-2115-3p) using the miRTarBase database (Supplementary Figure 2). We then investigated the mRNA expression of the predicted target genes using KEGG pathways analysis. The results showed that the mRNA targets of the differentially expressed miRNAs were enriched for the Oxytocin (OT) signaling pathway among the top 10 KEGG pathways (Supplementary Figure 3). GO enrichment analysis provides results in three categories: molecular function, cell component, and biological process. The top 10 GO terms in the three categories are shown in Supplementary Figure 3. We also found that insulin-like growth factor 1 receptor (*IGF-1R*), Ras-related C3 botulinum substrate 1 (*Rac1*), and mitogen-activated protein kinase 1 (*MAPK1*) were candidate target genes for the top miRNA miR-450a-2-3p (Supplementary Table 5).

### Mediating effect of EV miR-450a-2-3p on childhood trauma and adolescent MDD

The mediation models of EV miR-450a-2-3p and the path coefficients are shown in Table 2. The path analysis results of the trauma-miRNA-MDD chain were as follows: (1) total childhood trauma, emotional abuse, and physical neglect were associated with the miR-450a-2-3p gene (*p* < 0.05); (2) the miR-450a-2-3p gene was associated with adolescent MDD after adjustment for total childhood trauma, emotional abuse, or physical neglect (*p* < 0.05); and (3) total childhood trauma, emotional abuse, and physical neglect were directly associated with adolescent MDD after adjustment for the miR-450a-2-3p gene (*p* < 0.01). Moreover, the 95% CI of the mediation role of miR-450a-2-3p gene did not include zero through the bootstrap approach, which revealed that EV miR-450a-2-3p had significant mediating effects on the relationships between total childhood trauma, emotional abuse, physical neglect, and MDD. The standardized mediating effects of childhood trauma total, emotional abuse, and physical neglect on adolescent MDD through the miR-450a-2-3p gene were 0.290 (bootstrap 95%CI: 0.016-1.356, mediating effect accounted for 8.55% of the overall effect), 0.264 (bootstrap 95%CI: 0.021-1.105, mediating effect accounted for 10.51%), and 0.379 (bootstrap 95%CI: 0.058-1.243, mediating effect accounted for 24.66%), respectively.

### Diagnostic model for adolescent MDD based on EV miRNA and childhood trauma

**FIGURE 4:**
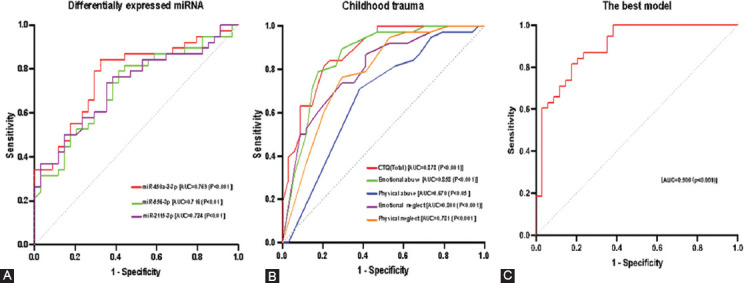
Receiver operator characteristic (ROC) curve was applied to examine the predictive ability of EV miRNA and childhood trauma for the risk of MDD development in adolescents. (A) The area under curve (AUC) for three differentially expressed EV miRNAs; (B) the AUC for childhood trauma total and different subtypes of trauma; and (C) the best model was composed of miR-450a-2-3p and emotional abuse. The AUC is reported as a measure of predictive accuracy. EV: Extracellular vesicles; miRNAS: microRNAs; MDD: Major depressive disorder.

To evaluate the accuracy of the diagnostic model of EV miRNA and childhood trauma on adolescent MDD, ROC curve analysis was applied to all patients with MDD and control subjects. The results revealed that, of the three differentially expressed EV miRNAs, miR-450a-2-3p (AUC: 0.769, 95%CI: 0.659~0.879) was able to diagnose adolescent MDD better than miR-556-3p (AUC: 0.716, 95%CI: 0.598~0.834), and miR-2115-3p (AUC: 0.724, 95%CI: 0.607~0.841) (Figure 4). For the diagnosis of adolescent depression, the best cutoff for miR-450a-2-3p relative expression was 1.916 with a sensitivity of 67.6% and a specificity of 84.2%.

We also observed that, of the different types of childhood trauma, emotional abuse could diagnose adolescent MDD with higher accuracy (AUC: 0.858, 95%CI: 0.767~0.948) than physical abuse (AUC: 0.670, 95%CI: 0.542~0.798), emotional neglect (AUC: 0.800, 95%CI: 0.698~0.902), and physical neglect (AUC: 0.781, 95%CI: 0.673~0.890) (Figure 4). The optimal cutoff of the emotional abuse score was 8.5, with a sensitivity and specificity of 82.4% and 78.9%, respectively. In addition, the total childhood trauma score had the best diagnostic ability of all the diagnostic factors with an AUC of 0.878 (95%CI: 0.799~0.956) (Figure 4). When the best cutoff value was 39.5, the sensitivity and specificity for diagnosing MDD were 79.4% and 81.6%, respectively.

We sought to recognize the best-fit integrated diagnostic factors for adolescents with MDD using a forward step-wise method of logistic regression. We evaluated the four differentially expressed miRNAs and different subtypes of childhood trauma. When we considered collinearity across all variables, the optimal model was composed of miR-450a-2-3p and emotional abuse, with an AUC of 0.900 (95%CI: 0.830~0.971, *p* < 0.001), the sensitivity and specificity for the diagnosis of adolescent MDD were 82.4% and 81.6%, respectively (Figure 4).

## DISCUSSION

To the best of our knowledge, this is the first research on the association between serum EV miRNA expression and adolescent MDD using high-throughput sequencing and quantitative RT-PCR. In the discovery stage, genome-wide miRNA expression profiling revealed that 32 miRNAs were differentially expressed between adolescent MDD patients and HC subjects. These included 18 upregulated and 14 downregulated miRNAs. Eight miRNAs with TPM greater than 10 were used for subsequent RT-PCR confirmation. We further confirmed that the expression of five EV miRNAs in adolescents with MDD was significantly higher than in HC and four of these were consistent with the expression patterns detected in the discovery stage. As the expression of miR-5100 was not confirmed by the RT-PCR verification, the gene was excluded from further analysis. After the Bonferroni correction, three miRNAs (miR-450a-2-3p, miR-556-3p, and miR-2115-3p) were still found to be significant and we found they were significantly associated with adolescent MDD after adjustments for demographics and lifestyle. Then, we conducted PCA on the 3 miRNAs and found that 95.62% of the variance was explained by PC-1. The clinical correlation analysis showed a positive correlation between PC-1 and clinical symptoms, and a negative correlation between PC-1 and the global function level. Simultaneous elevation in the expression levels of these three differentially expressed miRNAs was observed and there was collinearity between them. We speculate that EVs may deliver multiple miRNA molecules to cells simultaneously. To better understand the mechanism of the role of these three differentially expressed miRNAs in depression, we employed the miRTarBase (database based on experimental verification) [41] to predict the mRNA genes of miRNAs and performed pathway enrichment analysis. The results indicated that the three differentially expressed miRNAs were significantly abundant in the OT signaling pathway, which is known to be closely associated with MDD risk and suicide risk [42].

OT is a neurohormone involved in the regulation of mood and the OT signaling depends on the concentration of OT and the distribution of the OT receptors (OXTR). OT influences an individual’s sensitivity to social environment by increasing the salience of social cues [43]. It had also been found that the early alteration in OT signaling may disturb neuronal maturation and OXRT can be related to the regulation of synaptic scaffolding proteins [44]. Adolescence is a critical period for brain development and is characterized by continuous neural system maturation. EVs are important mediators for the transmission of signals between neural cells and play major roles in the neuronal stress response, neurogenesis, and synaptic plasticity processes in the central nervous system [45]. Given these features, we hypothesize that EV-mediated transmission of miRNAs results in changes in the expression of intracellular miRNAs that disrupt the normal transcription of target genes, which, in turn, cause alterations in neural mechanisms, ultimately increasing susceptibility to MDD in adolescents. Taken together, our findings reveal, for the first time, the existence of abnormal miRNA expression in EVs of adolescents with MDD. This suggests the importance of recognizing the role of molecular mechanisms, particularly the OT signaling pathway, in the development of MDD in adolescents.

The EV miR-450a-2-3p was chosen as a significant gene for further analysis as it showed the greatest level of change in the differential expression. Moreover, the gene also showed a significant ability to diagnose adolescent MDD, with a sensitivity of 67.6% and a specificity of 84.2%. MiR-450a-2-3p is a member of the miR-450a family. Recent studies showed increased expression of miR-450a in both skin and fibroblasts in rat models of depression [46]. They also found that miR-450a was present in both stress models (the ACTH and the CMS model), which suggested that miR-450a may affect the development of MDD through environmental stress-related pathways. In addition, Garbett et al. [47] observed elevated expression of miR-450a in the fibroblasts of patients with MDD. Importantly, these findings are consistent with our results, implying that the miR-450a gene family may be an important candidate target for MDD. While the miR-450a gene family has many members, our results indicate that miR-450a-2-3p is a promising biomarker for the diagnosis of MDD in adolescents. However, the specific functions of miR-450a-2-3p in neurobiology are currently unknown. We performed bioinformatic predictions on miR-450a-2-3p and found that it can target the regulation of the *IGF-1R* [48,49], *Rac1* [50], and *MAPK1* genes [51], which are known to be associated with MDD. We also found that these three target genes were mainly enriched in neurodevelopment, neuroinflammation, neurogenesis, and synaptic plasticity-related signaling pathways, including the MAPK, Neurotrophin, Ras, and PI3K-Akt signaling pathways [52-56]. Importantly, it has been shown that neurodevelopment, neuroinflammation, neurogenesis, and synaptic plasticity have key roles in the pathogenesis of MDD in adolescents [57-60].

A recent report described the upregulation of miR-139-5p in serum EVs in adult patients with MDD and showed that the miRNA was involved in the pathogenesis of MDD through the regulation of neurogenesis in the hippocampus [27]. Another study reported significant alterations in multiple serum EV miRNAs in a stress rat model of depression [61]. In addition, Li et al. [62] also identified two plasma EV miRNAs that were associated with treatment-resistant depression in a small sample of adults. These findings all suggest an essential role of EV miRNAs in the etiology of MDD. However, we did not identify the same miRNA from EVs in adolescent MDD. There could be three reasons for this observation. First, MDD is a clinically heterogeneous and complex disease, with diverse causes. Second, the sources and cargoes of EVs in the blood are very complex. Finally, we investigated adolescent population, whereas the most previous studies used adult samples. Although there are no diagnostic differences between adolescent and adult MDD, there are differences in clinical manifestation [63], risk factors [64], and antidepressant treatment response [65], implying the existence of different pathological mechanisms.

The previous studies have shown that early life stress, particularly childhood trauma, exerts a critical influence on the development of MDD in both adolescents and adults [66,67]. The theory of epigenetics suggests links between the environment, genes, and disease. Early life stress leaves an “imprint” on genes that can result in persistent alterations in transcription resulting in long-lasting effects on behavioral phenotypes and brain functions, increasing the individual’s vulnerability to developing depression later in life [16,68,69]. There is increasing concern regarding the relationship between early socioeconomic status and health and cognitive function in children and adolescents. Recent research from the Adolescent Brain Cognitive Development (ABCD) Study found that various factors reflecting socioeconomic status (including parent education, income, and neighborhood disadvantage) have different effects on the Resting State Functional Connectivity [70]. The previous studies [14] have found that children who grew up in poor environments had higher levels of methylation in the region of the *SLC6A*4 gene than those from wealthy families, which may lead to the lower serotonin levels in their brains. This was directly linked to the incidence of depression and the activation of the amygdala in poor children. This suggests that socioeconomic status may affect brain development and cognition through epigenetic mechanisms. Nevertheless, modulatory interactions between different environments may also affect the cognitive and mental health of adolescents. For example, a study from the Adolescent Brain Cognitive Development (ABCD) Study recently found that positive home and school environments can regulate the relationship between neighborhood disadvantage and brain functional connectivity, and positive environments can cushion the effects of neighborhood disadvantage on the cognitive and mental health of adolescents [71]. A recent study found that early life stress alters the expression levels of miR-449 and miR-34 in sperm samples from both male mice and humans, which are transmitted to the offspring [72]. A line of evidence has suggested that miRNA genes can serve as mediators to regulate the relationship between early life stress and MDD in adolescents and adults [73]. Our results showed that miR-450a-2-3p in EVs partially mediated associations between total childhood trauma, emotional abuse, physical neglect, and adolescent MDD. However, the mediating effects of miR-450a-2-3p were not the same for the three trauma types. The most significant effect of miR-450a-2-3p was seen in the influence of physical neglect on MDD, where a mediating ratio of 24.66% was obtained. Statistically, our results also confirmed a close relationship between stress, miRNA, and MDD. Experimentally, growing evidence indicates that early or chronic stress induces alterations in the concentrations of specific miRNAs expressed in the brain [74,75]. Changes in specific miRNAs in the brain were reported to be associated with depressive-like behaviors in stressed adolescent rats [15,76]. Considering these relationships, we used the different miRNAs and subtypes of childhood trauma to construct diagnostic models for adolescents with MDD. Our findings showed that the combination of miR-450a-2-3p and emotional abuse was the best model, with an AUC of 0.900 (sensitivity = 82.4% and specificity = 81.6%). In our opinion, this result is reasonable, because the increased morbidity of MDD in adolescents is related to the interaction of several factors (including the environment and genetics), and it is difficult to evaluate any single factor individually to effectively diagnose the disease [1]. The significance of this result lies in the identification of a more specific integrated diagnostic model based on psychosocial and biological factors.

There are several limitations to be mentioned here. First, we had a small sample size, so the possibility of false negatives cannot be excluded from the study. Second, the typical structure of EVs was not observed under TEM; this was likely due to the method used for EV extraction. Although ultracentrifugation is the classical method for EV extraction, it is time-consuming and often results in EVs with low purity which are easily damaged. We used an EV Isolation Kit based on a method using precipitation with polyethylene glycol. This method is commonly used in EV studies, second only to ultracentrifugation. It has many advantages, including being simple and fast, resulting in minimal damage to EVs. In addition, it can process many samples at the same time. Although the sizes of the EVs isolated by this method are the same as those separated by ultracentrifugation and ultrafiltration, the number of EVs and RNAs is significantly higher [77]. Third, we have not been able to determine the origin of the EVs isolated from serum. Although EV pellets are able to cross the blood-brain barrier [78], the fraction of neuron-derived EVs in EV pellets from the peripheral blood remains unknown, and further studies are required to verify that molecules present in the peripheral blood represent CNS abnormalities. Certainly, for insights into the pathophysiology of neuropsychiatric disorders, neuron-derived EVs would be more applicable. Although it has been shown that alterations in the size and miRNA cargo of neuron-derived EVs are correlated with MDD and antidepressant drug response [79] and some studies have extracted neuronally-enriched EV miRNAs from serum [80], the specific capture of neuronally derived EVs from peripheral blood is still technically difficult. Importantly, EVs in the peripheral blood are released, at least in part, through the nervous system [21,81]. Fourth, there are proteins or other types of RNA in EVs that we did not detect. Indeed, EVs carry other important cargoes, such as proteins, which have also been implicated in MDD or antidepressant treatment [82,83]. It may be possible to use a combination of kit isolation and immune-magnetic techniques to extract neuron-derived EVs, which may also assist with the identification of other types of EV cargo. Fifth, the expression pattern of one miRNA (miR-5100) differed between the high-throughput sequencing and quantitative RT-PCR. We speculate that the differential expression of miR-5100 observed in the discovery set was insufficiently significant, thus the difference cannot be shown stably in the validation set. In addition, the small sample size may have partly contributed to the inconsistency between the high-throughput sequencing and quantitative RT-PCR results. Finally, as this is a cross-sectional study, it is difficult to draw a causal inference. A larger sample size and follow-up studies would be needed.

## CONCLUSION

Our findings revealed significant changes in the expression levels of several miRNAs in the serum EVs of adolescents with MDD. We identified miR-450a-2-3p in EVs as a novel and important gene associated with MDD development in adolescents. Our results also confirmed the hypothesis that miRNAs serve as mediators between early stress and MDD development. Moreover, we also found that the combination of miR-450a-2-3p and emotional abuse was able to optimally diagnose MDD in adolescents. In view of the diverse etiology of depressive disorders, the development of an integrated diagnostic model combining psychosocial and biological factors may be a better strategy than using a single factor for the diagnosis of mental health problems. Overall, these findings shed a novel light on the understanding of the pathological mechanisms and diagnostic factors in the development of MDD in adolescents.
